# Service Use and Costs for People with Long-Term Neurological Conditions in the First Year following Discharge from In-Patient Neuro-Rehabilitation: A Longitudinal Cohort Study

**DOI:** 10.1371/journal.pone.0113056

**Published:** 2014-11-17

**Authors:** Diana Jackson, Paul McCrone, Iris Mosweu, Richard Siegert, Lynne Turner-Stokes

**Affiliations:** 1 King's College London, Faculty of Life Sciences & Medicine, Department of Palliative Care, Policy and Rehabilitation, London, United Kingdom; 2 King's College London, Institute of Psychiatry, Psychology & Neuroscience, Health Services and Population Research Department, London, United Kingdom; 3 AUT University, School of Public Health and Psychosocial Studies and School of Rehabilitation and Occupational Studies, Centre for Person Centred Research, Auckland, New Zealand; 4 Regional Rehabilitation Unit, Northwick Park Hospital, London, United Kingdom; Cardiff University, United Kingdom

## Abstract

**Background:**

Knowledge of the configuration and costs of community rehabilitation and support for people with long-term neurological conditions (LTNCs) is needed to inform future service development and resource allocation. In a multicentre prospective cohort study evaluating community service delivery during the year post-discharge from in-patient neuro-rehabilitation, a key objective was to determine service use, costs, and predictors of these costs.

**Methods:**

Patients consecutively admitted over one year to all nine London specialised (Level 1) in-patient neuro-rehabilitation units were recruited on discharge. They or their carers completed postal/web-based questionnaires at discharge and six and twelve months later, providing demographic data and measures of impairment, disability, service needs and provision. This paper describes health and social care service use, informal care and associated costs. Regression models using non-parametric boot-strapping identified predictors of costs over time.

**Results:**

Overall, 152 patients provided consistent data. Mean formal service costs fell significantly from £13,290 (sd £19,369) during the first six months to £9,335 (sd £19,036) from six-twelve months, (t = 2.35, P<0.05), mainly due to declining health service use. At six months, informal care was received on average for 8.2 hours/day, mean cost £14,615 (sd 23,305), comprising 52% of overall care costs. By twelve months, it had increased to 8.8 hours per day, mean cost £15,468 (sd £25,534), accounting for 62% of overall care costs. Being younger and more disabled predicted higher formal care costs, explaining 32% and 30% of the variation in costs respectively at six and twelve months.

**Conclusion:**

Community services for people with LTNCs carry substantial costs that shift from health to social care over time, increasing the burden on families. Prioritising rehabilitation services towards those in greatest need could limit access to others needing on-going support to promote their independence and reduce their reliance on families. This argues for greater investment in future rehabilitation services.

## Introduction

People with complex neurological conditions who are discharged from in-patient care often have on-going physical and psycho-social disabilities, and community rehabilitation services are best placed to address these. There is growing evidence worldwide for the effectiveness of community based neuro-rehabilitation in this group [1]. Gains in functional outcome, community integration and well-being have been demonstrated in stroke patients following physiotherapy interventions [2], [3]. And similar gains have been shown in patients with moderate to severe traumatic brain injuries after multi-disciplinary rehabilitation targeted towards improving hidden disabilities, such as cognitive and other aspects of psychological functioning [4], [5]. However, much less is known about patient contact with community health and social services, and what these cost to provide, as compared to in-patient services, which may partly reflect the wider diversity of community models of rehabilitation worldwide.

Community rehabilitation services are not always easy to access. They include health care delivered by medical and allied health professionals and social care, which provides home based personal care, day or residential care, equipment, advocacy and vocational support. These services vary considerably. For example, some are goal directed short interventions while others provide support over a longer period. Moreover, the nature and severity of patients' disabilities are likely to determine whether patients need programmes that focus predominantly on physical/functional re-education [3], cognitive/neurobehavioural interventions [6] or a combination of the two [7].

In the UK, the National Service Framework (NSF) for Long-Term Neurological Conditions (LTNCs) [8] was launched in 2005 with the aim of driving up standards of care for people with a range of LTNCs and their family carers. A key recommendation of the NSF was for better provision of community-based neuro-rehabilitation services to support improved community integration and participation.

Although National Health Service (NHS) spending on LTNC services has risen since 2005, with a reported 38% increase of £2.1 billion in 2006–07 to £2.9 billion in 2009–10 [9], the current configuration and costs of different models of community rehabilitation, how costs vary over time, and the predictors of these costs for people with LTNCs, are still unclear [10]. Determining service use and costs at a time of cost containment is important because this information can increase understanding of how resources are spread across people with disabilities of varying types and severities, identify shortfalls in provision and inform future resource allocation in response to assessed needs [11].

A number of cross-sectional studies report annual cost estimates of health services in the context of individual LTNCs and other long term chronic illnesses [12]–[14]. Others have investigated overall service costs for up to three years post onset [15], [16]. Cost data on formal and informal service provision in community dwelling adults with LTNCs covering nine diagnostic conditions, on average twelve years since condition onset, showed that both health and social care costs were higher for more dependent patients [14]. However, knowledge of how services and their costs vary longitudinally over the first critical year after discharge from specialist neuro-rehabilitation units, when patients might still be expected to gain independence in line with multi-disciplinary rehabilitation inputs is lacking. The transition from in-patient to community care entails significant adjustment on the part of patients and their families, and post-discharge support for injury-related deficits and emotional and psychosocial issues plays a vital part in re-establishing autonomy and social integration [17].

The health economic analysis presented here arose from a multicentre prospective cohort study that used a survey methodology to evaluate the community neuro-rehabilitation services received by patients with complex LTNCs who had been discharged from specialist in-patient rehabilitation services in London [18], [19]. This particular group of patients were chosen as they represented a population of people with complex disabilities, of whom a high proportion were expected to have on-going needs for rehabilitation and support.

Service use and costs were estimated in two ways. A new brief pragmatic instrument, the Needs and Provision Complexity Scale (NPCS), measured broad domains of service provision in relation to needs using a generic computerised costing algorithm [11]. These needs had been assessed formally by clinicians at the point of discharge from specialist in-patient rehabilitation units, and the best service provision that could be organised for each individual in accordance with their needs had been instigated. Findings six months later highlighted a number of gaps in service provision; most notably rehabilitation, social support and provision of equipment. The current analysis used data collected using the Client Service Receipt Inventory (CSRI) [20] to measure individual service use and costs more precisely, and also examined change in these variables over time. Our objectives were to determine the cost of different components of these services during the first and second six-month periods following discharge, and to identify the demographic and clinical factors, and measures of impairment and disability, that predicted service costs over time.

## Methods

### Study setting and participants

Within the London region, a consortium of nine tertiary (Level 1) in-patient rehabilitation units (the London Specialised Neuro-Rehabilitation Consortium (LSNRC)) provides specialist rehabilitation for patients with complex needs for rehabilitation that are beyond the scope of their local services. Patients represent a range of LTNCs and typically have physical problems, cognitive/behavioural/communication problems or various combinations of these. They often have ongoing needs for integrated care planning, multi-disciplinary rehabilitation and vocational support. A network of community based rehabilitation services provides on-going input after discharge to the community. However, community-based service provision is known to be patchy, and many patients report a fall-off in rehabilitation and support after they leave the in-patient services.

### Cohort study – whole sample

Consecutively admitted patients with LTNCs, who were subsequently discharged from all nine LSNRC units during a 12-month period spanning 2011–2012, were eligible for inclusion unless they (or their carer/proxy) declined to participate. Where cognitive/communication problems prevented patients from completing questionnaires, a family member or carer was identified to assist them wherever possible. Eligible patients or carers were given a verbal explanation about the study, provided with an information sheet and given the opportunity to ask questions. If they agreed to take part, consent or assent as appropriate was taken by the discharging clinician.

### Data collection

Data were collected at or soon after discharge, and at six and twelve months post-discharge, by means of postal or web-based questionnaires according to patient/carer choice. Demographic and diagnostic information, measures of impairment and disability, details of formal service use and informal care time were rated by patients/carers. Throughout the study, follow-up telephone interviews were used to support those who found questions difficult to answer, and to fill in any gaps in order to maximise the completeness of data collection. High attrition rates are well recognised in this patient population [18]. During the study, the full sample of 428 returned a total of 658 questionnaires over the three time points. The causes of attrition included patients who died, patients who changed their minds about participation and patients who failed to return questionnaires for no known reason [19]. The sub-set of 152 described in this paper had returned questionnaires at all three time points.

### Subset for longitudinal analysis

Patients were included in this longitudinal analysis if they had returned questionnaires at both six and twelve month time points and had consistent data on impairments, service use and costs.

Approval for the study was granted by Bromley Research Ethics Committee (Ref: 09/H0805/25) and covered all nine participating hospital sites. Local R&D consent was subsequently granted by internal clinical and research governance leads at The North West London Hospitals NHS Trust, King's College Hospital NHS Foundation Trust, South London & Maudsley NHS Foundation Trust, University College London Hospitals NHS Foundation Trust, Homerton University Hospital NHS Trust, St George's Healthcare NHS Trust, Central London Community Healthcare NHS Trust, The Royal Hospital for Neuro-disability and Blackheath Brain Injury Rehabilitation Centre & Neurodisability Service.

### Measures of outcome

#### Neurological Impairment Scale (NIS)

This brief 17-item checklist covers the major neurological impairments (motor/sensory loss, perception, speech and language, cognition, behaviour, mood, vision and hearing) that make up a complex presentation in people with LTNCs [21]. Total scores range from 0 (no impairments) to 50 (maximum impairments) and the tool has good scaling properties as an ordinal measure of impairment severity in two principal domains, ‘physical’ and ‘cognitive/behavioural’ impairment. This measure was completed for the recruited sample by LSNRC clinicians at discharge.

#### Northwick Park Dependency Scale (NPDS) and Care Needs Assessment (NPCNA)

The NPDS-CNA is a measure of nursing dependency and special nursing needs that records the time taken and number of people required to help with a wide range of daily activities [22], [23]. The full scale ranges from 0–100, but can be divided into two main sections. The basic care needs (NPDS-BCN) section has 16 items with a score range of 0 (independent) to 65 (dependent) and the special nursing needs section (NPDS-SNN) includes seven items indicating the need for nursing care, range 0–35. The NPCNA applies a computerised algorithm to the NPDS data in order to produce an estimate of the care hours per week needed by each individual in the community [23].

The NPDS-BCN section is further subdivided into two domains:

- NPDS Physical dependency domain (NPDS-PD) - 13 items (range 0–52) addressing needs for physical assistance, (binary rating: Largely independent  = 0–10, Dependent  = 11–52)
- NPDS Cognitive/behaviour dependency domain (NPDS-CB) - 3 items (range 0–13) addressing needs for support with communication, behaviour and safety awareness, (binary rating: Largely independent  =  Scoring 0–1 on all three items, Dependent  =  Scoring ≥2 on one or more items)

Because the severity and combination of physical and/or cognitive/behaviour impairments were expected to provide better indicators of support needs than diagnostic labels, adults were categorised into four dependency groups based on their binary NPDS-BCN scores in these two domains [14].

- Mild dependency: NPDS-PD and NPDS-CB  =  Largely independent
- Physical dependency: NPDS-PD  =  Dependent; NPDS-CB  =  Largely independent
- Hidden dependency: NPDS-PD  =  Largely independent; NPDS-CB  =  Dependent
- Mixed dependency: NPDS-PD and NPDS-CB  =  Dependent

#### Needs and Provision Complexity Scale (NPCS)

At set time points the NPCS was used to record (a) clinician rated needs for community health and social care services and (b) patient/carer rated provision of these services. An algorithm can be applied to these ratings to estimate the cost of packages of care for the purpose of integrated care planning [11]. The NPCS has acceptable reliability (in the context of this self-report measure, test-retest repeatability). Factor analysis suggests a scale structure in two principal domains, ‘Health and personal care’ and ‘Social care and support’, but also indicates a single general factor underpinning the full scale, with good overall internal consistency [24].

#### Client Service Receipt Inventory (CSRI)

A version of the Client Service Receipt Inventory (CSRI) specifically adapted for people with LTNCs was used to elicit data on all health and social care used by respondents over a defined period via interview or postal questionnaire [20]. In this case, patients/their carers recorded services received during the previous six months at (a) six months and (b) twelve months after discharge from LSNRC units. Information was collected on hospital in-patient stays and residential care, contact with day care and community services, hospital out-patient appointments, contact with primary and secondary healthcare professionals, as well as services received by respondents at home. Participants also estimated the number of hours per week that family members spent providing Informal care, such as help with self-care at home and/or support with social activities in the community, to supplement services received from outside agencies. Pro-rata adjustments were made where care had been received for only part of the period in question.

### Costs

Total health and social care costs across the two six monthly periods; that is 0–6 and 7–12 months post discharge, were estimated by combining service use data with appropriate national unit costs from recognised sources [25], [26] calculated in 2011/12 prices. We used the replacement cost method to value informal care time [27], where the cost of a paid professional at the time of data collection (2011/12); that is £18/hour of weekday contact from a Local Authority home care worker [25], was used as a ‘shadow price’ for informal care.

### Statistical analyses

Oneway analysis of variance with Bonferroni corrections for multiple comparisons was used to analyse change in dependency scores over time during the year following discharge. In fourteen instances, participants had entered clearly mistaken service use data for the number and/or duration of contacts with either a doctor, therapist or home care worker. These data were replaced with median imputations from other valid cases for each relevant service, which were derived from (a) total service contacts and (b) duration of services used by patients across the sample. Paired t-tests were used to compare the mean costs of both informal and formal service use between the two six-month time periods for (a) users of each service and (b) the full sample. Further cost comparisons over time were examined by gender, diagnostic group and dependency group, and Pearson's correlation coefficients were computed to examine associations between costs and age, impairment and dependency measures.

Predictors of formal costs over time were identified using regression models with cost as the dependent variable and characteristics of patients as independent variables. Because cost data usually have a skewed distribution due to a small number of patients having disproportionately high costs, stepwise linear regression models were used with confidence intervals around coefficients produced using non-parametric bootstrapping. This widely used Monte Carlo approach entails repeated sampling with replacement from the sample [28]. Variables included in these models were gender, age, NIS motor and cognitive impairments, NPDS total score, mild, physical, hidden and mixed dependency groups and NPCS needs. These were retained or discarded based on a significance level of P<0.05. Data were analysed using Stata 10.1 [29].

## Results

### Study participants

A sub-set of 152 (36%) out of the full sample of 428 patients recruited to the cohort study [18] returned questionnaires at both six and twelve month time points with consistent data on impairments, service use and costs, and were included in this longitudinal analysis. The demographic and clinical characteristics, discharge destination and referral to services for these two groups are set out in Table 1, which shows virtually no differences between them.

**Table 1:** Demographic and clinical characteristics rated at or soon after discharge for participants in the cohort study whole sample (N = 403–428) as compared to the sub-set included in the longitudinal analysis (N = 152).

Variables	Cohort study whole sample (N = 428)		Sub-set for longitudinal analysis (N = 152)	
	Mean	(SD)	Mean	(SD)
Age (years)	49.1	(15.3)	50.1	(14.1)
NIS motor score	8.1	(5.1)	8.1	(5.2)
NIS cognitive score	4.5	(3.1)	4.3	(3.2)
NIS total	12.8	(6.4)	12.8	(6.4)
NPDS total*	12.7	(13.9)	12.7	(13.3)
NPCS-Needs	17.7	(7.8)	17.2	(7.8)
**Gender**	**N**	**(%)**	**N**	**(%)**
Male	270	(63%)	96	(63%)
Female	158	(37%)	56	(37%)
**Diagnosis**				
CVA/stroke	212	(50%)	79	(52%)
Traumatic brain injury	63	(15%)	18	(12%)
Other acquired brain injury	40	(9%)	13	(9%)
Spinal cord injury	38	(9%)	11	(7%)
Peripheral neuropathy	26	(6%)	8	(5%)
Progressive LTNC	21	(5%)	7	(5%)
Other	27	(6%)	16	(10%)
Missing	1	(0.2)		
**Discharge destination**				
Home	339	(79%)	125	(82%)
Nursing home	52	(12%)	13	(9%)
Residential rehabilitation	15	(4%)	7	(5%)
Hospital for other reasons	4	(1%)	2	(1%)
Other	18	(4%)	3	(2%)
**Referral to services**				
Not referred for rehabilitation	40	(10%)	15	(10%)
Referred for on-going rehabilitation#	378	(90%)	137	(90%)
**Type of on-going rehabilitation**				
Community rehabilitation team	297	(69%)	108	(71%)
Other in-patient rehabilitation	25	(6%)	10	(7%)
Out-patient or home therapy services	46	(11%)	21	(14%)
Vocational rehabilitation	31	(7%)	8	(5%)
Other rehabilitation	56	(13%)	20	(13%)

NIS  =  Neurological Impairment Scale, NPDS  =  Northwick Park Dependency Scale NPCS  =  Needs and Provision Complexity Scale.

* Completed soon after discharge by N = 256 across the whole sample and N = 135 across the sub-set for longitudinal analysis.

# Some patients received more than one service.

Patients in the sub-set were of average age 50 years, males outnumbered females by 2∶1 and three-quarters had sustained a stroke or other brain injury. The majority returned home after discharge and most were referred for on-going rehabilitation; 108 (71%) of them to a community rehabilitation team. Others were referred to an in-patient or other rehabilitation provider, out-patient therapy services, or to a combination of services. Fifteen patients were not referred to specific services at the time of discharge but accessed them at a later date. Overall levels of dependency rated soon after discharge ranged from 0–66, mean (sd) 12.7 (13.3) and did not change significantly during the year post discharge.

### Service use and costs

The total cost of care (formal and informal) for the full sample averaged £27,905 (sd £29,574) across the first six monthly period, and by twelve months had reduced, but not significantly, to an average of £24,803 (sd £30,515). Table 2 summarises formal service use and costs between 0–6 and 7–12 months for participants using each service (singly and in combination), and per capita for the full sample of 152. Formal costs averaged £13,290 (sd £19,369) during the first six months, making up 48% of total costs, but by twelve months had fallen significantly to a mean of £9,335 (sd £19,036); (t = 2.35, P<0.05) and made up 38% of total costs. It is not uncommon for health economic data to be skewed and the high standard deviations found in our formal costs data reflect this. These data were further skewed by a few outliers who used costly in-patient or residential services, or who required two carers round the clock, including waking night care, which in the community would often entail employment of a team of six whole time carers working in shifts.

**Table 2:** Formal and informal service use and costs (2011/12 £s) during the two six-monthly periods following discharge for users of each service and the full sample (N = 152).

	Service users				Contacts by users				Costs per user				Costs full sample			
Service type	0–6 months		7–12 months		0–6 months		7–12 months		0–6 months		7–12 months		0–6 months		7–12 months	
	N	(%)	N	(%)	Mean	(SD)	Mean	(SD)	Mean	(SD)	Mean	(SD)	Mean	(SD)	Mean	(SD)
**Health services**													11,069	(28,263)	**4,828**	**(17.126)** *
***Stays in ITU, neurology, medical, rehab and other wards***																
In-patient days	41	(27%)	**29**	**(19%)**	33.3	(48.5)	**17**	**(35.1)**	18,113	(25,911)	**9,491**	**(18,358)**	4,886	(15,585)	**1,811**	**(8,746)**
***Out-patient consultations with one or more health professionals***																
General practitioner	100	(66%)	**85**	**(56%)**	4.3	(3.8)	**3.6**	**(3.5)**	200	(181)	**173**	**(221)**	131	(174)	**97**	**(186)**
Neurologist	72	(47%)	**59**	**(39%)**	1.7	(1.0)	1.7	(1.1)	234	(187)	***277***	***(307)***	111	(174)	**108**	**(234)**
Rehabilitation doctor	28	(18%)	**11**	**(7%)**	3.1	(4.3)	**1.5**	**(0.5)**	586	(1,291)	**279**	**(209)**	108	(591)	**20**	**(90)**
Other doctor	45	(30%)	**38**	**(25%)**	2.8	(1.8)	2.8	(2.0)	468	(669)	**367**	**(500)**	139	(420)	**92**	**(294)**
Dentist	30	(20%)	**28**	**(18%)**	2.3	(1.4)	**1.7**	**(1.3)**	173	(103)	**126**	**(100)**	34	(82)	**23**	**(65)**
General practice nurse	17	(11%)	*23*	*(15%)*	3.1	(3.3)	**2.2**	**(1.6)**	34	(42.5)	**26**	**(26)**	4	(18)	4	(14)
Nurse specialist	12	(8%)	**8**	**(5%)**	2.1	(1.1)	***3.4***	***(3.1)***	67	(36.3)	***153***	***(146)***	5	(21)	***8***	***(46)***
Physiotherapist	50	(33%)	**38**	**(25%)**	12.7	(13.5)	***15.5***	***(16.4)***	1,459	(2,033)	***2,058***	***(3,240)***	480	(1,347)	***514***	***(1,836)***
Occupational therapist	32	(21%)	**15**	**(10%)**	7.0	(8.5)	**6.9**	**(8.4)**	721	(906)	**530**	**(586)**	152	(505)	**52**	**(239)**
Speech/language therapist	25	(16%)	**16**	**(11%)**	8.2	(8.4)	**8.1**	**(8.0)**	1,042	(1,107)	***1,175***	***(1,214)***	171	(587)	**124**	**(527)**
Psychologist	15	(10%)	**9**	**(6%)**	2.8	(1.8)	***7.8***	***(8.0)***	464	(425)	***1,441***	***(1,600)***	46	(190)	***85***	***(502)***
Counsellor	3	(2%)	3	(2%)	12	(7.2)	**3.3**	**(5.8)**	1,078	(533)	**275**	**(476)**	21	(162)	**5**	**(67)**
Mental health worker	1	(0.7%)	1	(0.7%)	16	(0)	**8**	**(0)**	1,216	(0)	**608**	**(0)**	8	(99)	**4**	**(49)**
***Home visits from one or more community health professionals***																
General nurse	18	(12%)	**11**	**(7%)**	5.4	(7.1)	***13.3***	***(19.1)***	554	(575)	***3,618***	***(7,547)***	66	**(264)**	***262***	*(2,158)*
Physiotherapist	39	(26%)	**27**	**(18%)**	5.6	(4.9)	**5.1**	**(2.8)**	2,828	(3,390)	***2,993***	***(2,224)***	726	**(2,104)**	**532**	(1,473)
Occupational therapist	35	(23%)	**16**	**(11%)**	3.7	(3.4)	**2.9**	**(3.0)**	1,885	(1,844)	**1,859**	**(2,432)**	434	**(1,183)**	**196**	(957)
Speech/language therapist	18	(12%)	**9**	**(6%)**	5.3	(4.5)	**3.9**	**(3.1)**	2,524	(2,724)	***3,065***	***(4,362)***	299	**(1,227)**	**181**	(1,239)

	Service users				Contacts by users				Costs per user				Costs full sample			
Service type	0–6 months		7–12 months		0–6 months		7–12 months		0–6 months		7–12 months		0–6 months		7–12 months	
	N	(%)	N	(%)	Mean	(SD)	Mean	(SD)	Mean	(SD)	Mean	(SD)	Mean	(SD)	Mean	(SD)
**Social services**													5,339	(12,059)	**5,166**	**(15,789)**
***Residential care and/or day care in hospitals/day centres***																
Residential care (days)	16	(11%)	16	(11%)	130.6	(73.6)	**123**	**(80.6)**	25,576	(14,825)	**23,347**	**(15,628)**	2,692	(9,157)	**2,458**	**(8,714)**
Day care	24	(16%)	**18**	**(12%)**	6.5	(4.1)	**5.2**	**(1.9)**	3,361	(2,125)	**2,802**	**(1,456)**	531	(1,483)	**332**	**(1,031)**
***Home-based visits from one or more social care staff***																
Social worker	16	(11%)	**7**	**(5%)**	1.9	(1.4)	***3.6***	***(2.8)***	1,956	(1,438)	***4,070***	***(3,275)***	206	(754)	**187**	**(1,076)**
Personal care	21	(14%)	**10**	**(7%)**	35.7	(23.6)	***50.8***	***(16.9)***	5,706	(5,407)	***8,922***	***(3,834)***	788	(2,788)	**587**	**(2,409)**
Domestic help	12	(8%)	***14***	***(9%)***	13.2	(12.0)	**11.5**	**(10.9)**	2,739	(2,948)	***3,660***	***(4,003)***	216	(1,087)	***337***	***(1,583)***
Day sitting service	4	(3%)	**3**	**(2%)**	7.8	(8.2)	***18***	***(13.1)***	9,401	(13,603)	***29,760***	***(33,064)***	247	(2,440)	***587***	***(5,633)***
Night sitting service	2	(1.3%)	**1**	**(0.7%)**	16.5	(19.1)	***30***	***(0)***	26,956	(32,901)	***66,960***	***(0)***	355	(4,082)	***441***	***(5,431)***
**Total formal costs**													13,290	(19,369)	**9,335**	**(19,036)**
**Combinations of services**																
Secondary care doctors	97	(64%)	**81**	**(53%)**	3.4	(13.5)	**2.8**	**(2.1)**	560	(923)	**412**	**(471)**	357	(784)	**220**	**(400)**
Out-patient therapists	67	(44%)	**49**	**(32%)**	16.0	(16.5)	***17.7***	***(19.0)***	1,838	(2,269)	***2,219***	***(3,172)***	810	(1,758)	**715**	**(2,069)**
Home-based therapists	57	(38%)	**36**	**(24%)**	7.3	(9.0)	**5.9**	**(4.9)**	3,890	(4,969)	**3,838**	**(4,181)**	1,459	(3,568)	**909**	**(2,595)**
Out-pt/home physiotherapy	76	(50%)	**58**	**(38%)**	11.2	(11.9)	***12.5***	***(14.2)***	2,411	(3,070)	***2,742***	***(3,041)***	1,206	(2,479)	**1,046**	**(2,297)**
Out-pt/home occ. therapy	56	(37%)	**27**	**(18%)**	6.3	(7.1)	**5.6**	**(6.7)**	1,590	(1,680)	**1,396**	**(1,972)**	586	(1,273)	**248**	**(978)**
Out-pt/home speech therapy	40	(26%)	**24**	**(16%)**	7.5	(7.2)	**6.8**	**(6.9)**	1,787	(2,096)	***1,932***	***(2,887)***	470	(1,326)	**305**	**(1,330)**
Rehab/therapy services#	104	(68%)	**72**	**(47%)**	-	-	-	-	8,022	(16,150)	**5,362**	**(12,221)**	5,489	(13,853)	**2,540**	**(8,800)**
Mental health services	23	(15%)	**15**	**(10%)**	5.7	(5.0)	***6.5***	***(6.7)***	1,026	(961)	***1,148***	***(1,338)***	155	(520)	**113**	**(533)**

* A fall in costs between 0–6 and 7–12 months is emphasised in bold while a rise is emphasised in bold italics. Costs that varied by 10% or less are not emphasised.

# Contacts by users comprised days in rehabilitation settings and out-patient or home visits from therapists so could not be totalled.

A quarter of the participants (N = 41) had received further in-patient treatment at some stage during the first six monthly period. On average the cumulative number of in-patient days exceeded one month, although this may have been due to more than one admission. In-patient care costs, at an average of £18,113 per person using these services accounted for 37% of total formal care costs. Two-thirds (66%) had contacts with general practitioners (GPs), but this service only made up 1% of overall formal care costs. Nearly half (43%) had contacts with neurologists and 18% saw a rehabilitation doctor in a community or out-patient setting. Out-patient therapy use was relatively high, being accessed by 44% of the sample for an average of 16 contacts per month, and accounting for 6% of formal care costs. However, only 12% had contact with a psychologist or counsellor. More than a third of the sample (38%) had home-based therapy, averaging 7 contacts per month, and costing more than twice as much as out-patient therapy. Just 11% of patients had contact with a social worker and 16% accessed day-care services. Around 14% received personal care and/or domestic help or other home care.

By twelve months, despite their level of dependency remaining broadly the same, participants' use of health and social care services had largely declined. Not only did fewer of them access services of almost every kind, but user contacts also diminished, notably those with some doctors, home-based therapists and residential and day care centres. The proportion having in-patient care decreased by a third and the number of days in hospital for those who were admitted fell by half, accounting for 19% of formal care costs. When rehabilitation services (comprising days in rehabilitation settings and contacts with community therapists) were singled out, a significant reduction in costs over time of 54% was found across the full sample (t = 2.89; P<0.01). In contrast, an increase in contacts over time (albeit by fewer participants) led to a rise in costs per service user accessing general and specialist nurses, out-patient physiotherapists, psychologists, social workers, home-based personal care and day/night sitting services.

### Informal service use and costs

**Figure 1:**
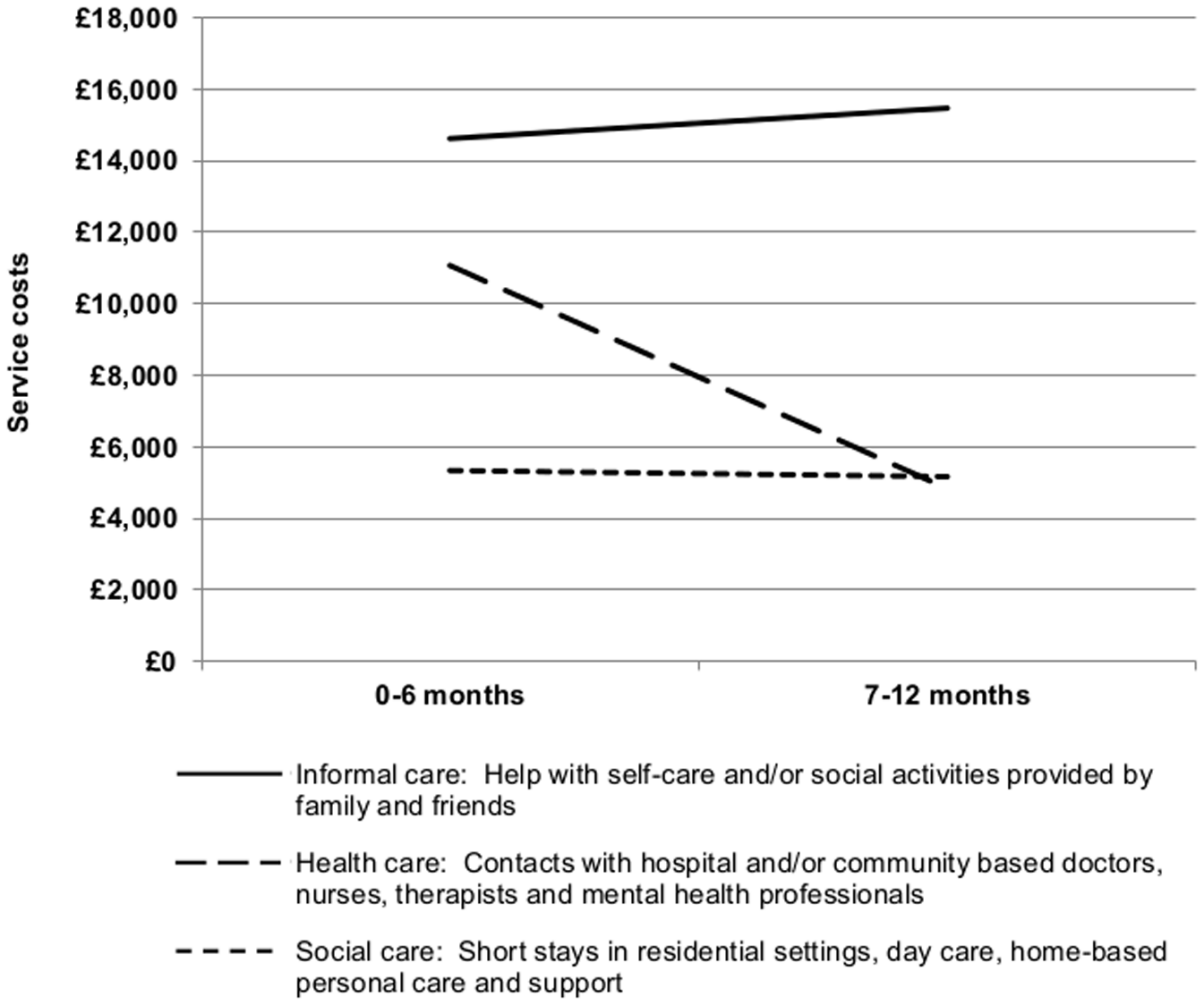
Change in mean service costs over time. Figure 1 shows a rise in the mean costs of informal care alongside a fall in the mean costs of both health and social care services over time. These services were received by patients with complex long term neurological conditions during the year following discharge from specialist in-patient neuro-rehabilitation services in London.

During the first six months after discharge, more than half the sample (57%) received informal care from family or friends for an average of 8.2 hours per day, at a mean (sd) cost of £14,615 (£23,305) and accounting for 52% of the overall cost of care in this period. During the second six months, the average duration of informal care increased to 8.8 hours per day at a mean (sd) cost of £15,468 (£25,534). Although informal costs did not differ significantly over time, by twelve months they accounted for 62% of the overall cost of care; 10% more than at six months post-discharge, suggesting that as formal care services declined, family and friends were spending more time supporting participants. Figure 1 highlights the increase in informal care costs over time, as compared to the decline in overall care costs of both health and social care services as set out in Table 2.

### Formal, informal and total costs by demographic and clinical characteristics

A breakdown of formal, informal and total costs over time by gender, diagnosis and dependency group, is set out in Table 3. With only a few exceptions, the trend for formal care costs to fall over time while informal care costs rose or remained broadly the same held for the sub-sets within variables. Exceptions comprised those with acquired brain injuries, spinal cord injuries and mixed dependencies, for whom both formal and informal costs fell over time. In contrast, formal costs rose over time for the few with progressive conditions, with a corresponding fall in informal care costs.

**Table 3:** Formal, informal and total care costs (2011/12 £s) overall and by demographic and clinical variables during the two six-monthly periods following discharge (N = 152).

			Formal care costs						Informal care costs						Total care costs					
			0–6 months		7–12 months				0–6 months		7–12 months				0–6 months		7–12 months			
	N	%	Mean	(sd)	Mean	(sd)	t	P	Mean	(sd)	Mean	(sd)	t	P	Mean	(sd)	Mean	(sd)	t	P
**Six monthly costs overall**	152	(100%)	13,290	(19,369)	**9,335**	**(19,036)** *	2.35	<0.05	14,615	(23,305)	15,468	(25,534)	−0.42	-	27,905	(29,574)	24,803	(30,515)	1.22	-
**Breakdown of six monthly costs by gender, diagnosis and dependency**																				
**Gender**																				
Male	96	(63%)	13,216	(20,694)	**8,402**	**(14,030)**	2.58	<0.05	14,674	(23,329)	14,326	(24,119)	0.13	-	27,890	(31,252)	**22,728**	**(27,651)**	1.56	-
Female	56	(37%)	13,418	(17,036)	**10,934**	**(25,510)**	0.76	-	14,512	(23,475)	***17,426***	***(27,910)***	−0.95	-	27,930	(26,724)	28,360	(34,863)	−0.11	-
**Diagnosis**																				
CVA/stroke	79	(52%)	11,762	(18,482)	**8,533**	**(21,562)**	1.17	-	16,509	(23,368)	***21,475***	***(29,146)***	−1.68	-	28,085	(29,134)	30,008	(35,197)	−0.44	-
Traumatic brain injury	18	(12%)	15,037	(25,152)	**12,275**	**(24,187)**	0.87	-	1,716	(4,910)	1,586	(4,152)	0.11	-	16,753	(24,941)	**13,861**	**(24,489)**	0.85	-
Other acquired brain injury	13	(9%)	21,902	(24,773)	**10,123**	**(15,905)**	2.37	<0.05	24,029	(32,726)	**12,246**	**(15,049)**	1.38	-	45,931	(40,759)	**22,369**	**(21,035)**	2.51	<0.05
Spinal cord injury	11	(7%)	22,253	(24,979)	**14,946**	**(14,467)**	0.92	-	12,949	(24,317)	**10,526**	**(23,130)**	1.09	-	35,202	(30,284)	**25,472**	**(26,560)**	1.19	-
Peripheral neuropathy	8	(5%)	4,448	(5,693)	**2,431**	**(4,115)**	0.74	-	5,219	(6,111)	***10,689***	***(27,544)***	−0.65	-	9,667	(10,562)	***13,120***	***(26,836)***	−0.39	-
Progressive LTNC	7	(5%)	11,476	(9,637)	***13,684***	***(11,809)***	−0.41	-	19,298	(27,024)	**3,472**	**(5,790)**	1.48	-	30,774	(25,972)	**17,157**	**(9,213)**	1.97	-
Other	16	(10%)	10,927	(11,651)	**7,034**	**(9,193)**	1.81	-	15,918	(26,141)	15,080	(26,326)	0.11	-	26,844	(26,764)	**22,115**	**(25,659)**	0.59	-
**Dependency group**																				
Mild	79	(52%)	8,121	(15,026)	**3,767**	**(5,459)**	2.54	<0.05	9,162	(17,510)	9,676	(20,662)	-0.22	-	17,284	(22,993)	**13,443**	**(21,480)**	1.48	-
Physical	19	(12%)	11,797	(11,985)	**9,878**	**(12,170)**	1.66	-	20,102	(26,373)	20,741	(26,207)	−0.11	-	31,899	(27,146)	30,619	(25,208)	0.21	-
Hidden	28	(18%)	17,262	(22,038)	**12,705**	**(19,779)**	1.26	-	14,730	(19,597)	***24,784***	***(30,807)***	−1.58	-	31,992	(28,415)	***37,490***	***(34,492)***	−0.69	-
Mixed	26	(17%)	25,810	(25,820)	**22,225**	**(35,818)**	0.48	-	27,047	(33,626)	**19,180**	**(29,115)**	1.71	-	52,292	(34,526)	**41,405**	**(39,268)**	1.41	-

* A fall in costs between 0–6 and 7–12 months is emphasised in bold while a rise is emphasised in bold italics. Costs that varied by 10% or less are not emphasized.

Associations between formal and informal costs during the first and second six-monthly periods, and continuous variables comprising age at discharge, impairments, clinician determined needs for services and disability at discharge and six months are shown in Table 4. Motor and cognitive impairments, needs for services and level of disability were significantly associated with formal costs at 0–6 months, with a trend for stronger associations at 7–12 months. Age was also negatively associated with formal costs at 7–12 months. In general, associations with informal care costs were not as strong during the first six months and notably weaker during the second six monthly period. These findings suggest a tendency for formal services to be targeted towards younger more disabled people over time.

**Table 4:** Correlation matrix (Pearson's r) showing associations between formal and informal costs during the two six-monthly periods following discharge and age, impairments, needs for services and disability (N = 152).

	Costs 0–6 months		Costs 7–12 months	
	Formal	Informal	Formal	Informal
Age	−0.16	0.02	−0.27**	0.13
NIS motor score	0.27**	0.21*	0.41***	0.16
NIS cognitive score	0.19*	0.24**	0.17*	0.17*
NPCS total needs	0.30***	0.16*	0.31***	0.12
NPDS total score at discharge	0.36***	0.26**	-	-
NPDS total score at six months	-	-	0.46***	0.09

*P<0.05,

**P<0.01,

***P<0.001.

Table 5 sets out a series of regression models identifying significant predictors of formal care costs for the two six-monthly periods. Age, motor impairments and dependency of varying kinds were the strongest predictors of costs. In Model 1, each year less in patients' age significantly increased formal costs by £249. And every point increase in NPDS score, indicating greater dependency, added £530 to costs. This model explained 15% of the variation in formal costs by six months. During the second six-monthly period, each year less in patients' age increased costs by £375 and every point increase in NIS motor score and NPDS score respectively added £821 and £496 to costs. This model explained 32% of the variation in costs from 6–12 months.

**Table 5:** Bootstrapped regression models to identify predictors of formal care costs for the two six-monthly periods following discharge (2011/12 £s).

Independent variables	0–6 months		7–12 months	
	β	95% CI	β	95% CI
**Model 1**				
Males^1^	-	-	-	-
Age	−249**	−435, −63	−375*	−674, −76
NIS motor	-	-	821*	91, 1551
NIS cognitive	-	-	-	-
NPCS needs	-	-	-	-
NPDS total#	530**	221, 838	496*	96, 895
**Adjusted R** 2	**15%**		**32%**	
**Model 2**				
Males^1^	-	-	-	-
Age	−208*	−409, −6	−324*	−587, −61
NIS motor	963**	381, 1545	1415**	357, 2473
NIS cognitive	-	-	-	-
NPCS needs	-	-	-	-
Physical dependency^2^	-	-	-	-
Hidden dependency^2^	8951**	1811, 16092	9243**	2289, 16197
Mixed dependency^2^	12083*	4298, 19868	12743*	1144, 24343
**Adjusted R** 2	**17%**		**30%**	

*significant at <0.05,

**significant at <0.01,

***significant at <0.001.

1 Compared to females,

2 mild impairments

# NPDS scores at discharge were used in the 0–6 month model and at six months in the 7–12 month model.

β is the value for predicting the dependent variable from the independent variable.

R^2^ is the proportion of variance in the dependent variable which can be explained by the independent variables and is adjusted to allow for extraneous predictors to the model.

When NPDS dependency groups were substituted for overall NPDS scores in Model 2, similar but more detailed findings emerged. By six months, each year less in patients' age increased costs by £208 and the trend for costs to increase in line with greater severity of motor impairments was also shown; with a one-point increase in NIS motor score adding £963 to costs. Patients with hidden dependency added £8,951, while those with mixed dependency added £12,083 to costs. This model explained 17% of the variation in costs from 0–6 months. Between 7–12 months, costs increased to £324 for each year less in patients' age; every point increase in NIS motor score added £1,415 to costs and patients with hidden dependency added £9,243 to costs, while those with mixed dependency added £12,743. This model explained 30% of the variation in costs at twelve months.

## Discussion

In this study we examined the costs of formal and informal services used by patients with complex LTNCs during the year following discharge from specialist in-patient neuro-rehabilitation units in London. As noted by others, some services were intensively used by small numbers of patients but had large proportions of non-users [30]. Even so, the numbers of patients accessing formal services of almost every kind tended to decline over time and aside from some therapy services, most health service costs fell between six and twelve months, with rehabilitation costs in particular falling significantly. In contrast, contacts with social care services and their associated costs increased substantially over time, as did the costs of informal care, indicating a shift from health to social care service use during the year following discharge, and an increase in the time that family carers spent supporting daily activities.

Despite rehabilitation/therapy inputs of varying kinds being received by two thirds of the sample for up to six months and by half for up to twelve months, dependency did not change significantly between discharge and follow-up at six and twelve month time points. This conflicts with evidence showing that increased therapy intensity in the context of integrated services providing on-going community support can accelerate the recovery of personal independence and enhance functional recovery in patients with strokes and brain injuries [6], [31], [32], [33]. There are several possible explanations for this finding. It could be that the inputs received by patients in this study were not sufficiently wide-ranging, intensive and prolonged to have had a significant effect on independence. Alternatively, that a high proportion of patients in this group with complex LTNCs were not expected to respond to rehabilitation inputs. Another possibility is that measurable gains in this group were likely to take longer than one year to achieve. This highlights the need for longitudinal research into service inputs and disability outcomes in these patients to increase understanding of their recovery patterns over time.

As formal health care costs reduced, so informal care costs tended to increase and this relationship varied by dependency group. The greatest significant drop in formal costs over time occurred in patients with mild dependencies, for whom there was a minimal change in informal care costs, implying that they had mostly achieved their rehabilitation goals by six months after discharge, but were nonetheless still requiring some degree of additional support from their families. In contrast, there was on average, a 26% drop in formal care costs during the second six months against a 68% rise in informal care costs for patients with hidden problems. These findings tend to support the view that limitations in resources rather than a lessening of need is the main driver of service reduction [34]. Comparatively few patients in this study had access to psychology and other mental health services. The shortage of these services for patients with challenging cognitive/behavioural disabilities has been highlighted in other studies [35], [36], as has the negative impact of these problems on family carers [37].

Formal service costs for those with mixed physical and hidden problems were greater than for those in other dependency groups, during both six-monthly periods, and remained broadly similar over time. These individuals had a range of disabilities and psychosocial problems, and were likely to need on-going access to integrated community-based health and social services to support their continued rehabilitation [5], [38]. This finding may reflect the ‘inverse therapy rule’, in that where resources are limited, services will be focused on those patients with the severest problems who have the greatest needs [18].

In this study, being younger and more severely disabled were predictors of higher formal service costs, explaining 32% and 30% of the variation in costs respectively at six and twelve months post discharge. A similar relationship between costs and disability over time has been found in the context of stroke [30], though other cited contributory factors, such as co-morbidities and place of residence, were not considered in this study, preventing direct comparison of findings.

There are a number of limitations to the analysis presented here. One important one being that needs were not re-assessed at six months. Therefore although we know that patients were still overall as dependent as they were at discharge, we do not know if the reduction in services represented reduced needs on a case by case basis or time-limited services in general.

The CSRI provides data on the types and duration of services used, but a range of other factors could also have impacted on service costs. For example, the provision of aids, equipment and adaptations to accommodation. These were included in analyses using the NPCS and are reported on elsewhere [11], but were not costed to supplement CSRI data and this should be borne in mind when making comparisons with the literature on LTNC care costs more generally.

Completion of the CSRI was dependent on the patient/family's recall of service use over the six months prior to each data collection point. This may be inaccurate, especially where patients themselves reported, as a number had some degree of cognitive/communication problems. However, telephone support from the research team at six and twelve months post-discharge helped to ensure completeness and accuracy of data. And as is common in health economic data, there were some outliers with disproportionately high estimated costs, largely accounted for by small numbers of patients accessing costly in-patient care services, residential care and day/night sitting services. These were addressed in part by boot strapping.

Different community rehabilitation programmes are likely to vary in terms of interventions offered. This study focused on costs and we did not attempt to measure aspects such as the precise content of interventions, the quality, intensity and skill mix of interdisciplinary team inputs, whether the rehabilitation programmes were tailored to patients' needs in the home environment, or the extent to which patients and families were empowered to assume responsibility for elements of rehabilitation. These have all been cited as having an influence on dependency and improved outcomes [31], [32], [39]. It would be informative to examine these variables in future studies of this kind.

This paper reported on all patients referred for on-going community neuro-rehabilitation. They included a wide range of conditions with very different support needs and prognoses - i.e. those with progressive conditions, sudden onset conditions, those with predominantly spinal or physical disabilities and those with predominantly behavioural, or cognitive or communication disorders. The results reflect the proportions of these different conditions in the sample, limiting generalisation of findings to other samples of people with neurological conditions in different proportions. However, the study was conducted in the context of real life clinical practice and our sample is likely to reflect to some extent the number and variety of conditions typically seen in the context of specialist community neuro-rehabilitation services designed to support patients with complex needs.

In conclusion, the provision of community services to support the on-going rehabilitation of people with complex LTNCs following discharge from in-patient care carries significant costs that shift from health to social care, and involves considerable input from family carers according to the nature and severity of patients' disabilities. Prioritising services towards those in greatest need could limit access to others needing on-going specialist community rehabilitation services, which may have implications for the independence and well-being of patients and their families more generally. Further methodologically sound research to increase knowledge of service inputs, outcomes and costs by condition type and disability over longer time scales is now needed to inform the provision of equitable high quality services that promote autonomy for people with complex LTNCs.
